# A Systematic Integration of Artificial Intelligence Models in Appendicitis Management: A Comprehensive Review

**DOI:** 10.3390/diagnostics15070866

**Published:** 2025-03-28

**Authors:** Ivan Maleš, Marko Kumrić, Andrea Huić Maleš, Ivan Cvitković, Roko Šantić, Zenon Pogorelić, Joško Božić

**Affiliations:** 1 Department of Abdominal Surgery, University Hospital of Split, Spinčićeva 1, 21000 Split, Croatia; 2 Department of Pathophysiology, School of Medicine, University of Split, Šoltanska 2A, 21000 Split, Croatia; 3 Laboratory for Cardiometabolic Research, School of Medicine, University of Split, Šoltanska 2A, 21000 Split, Croatia; 4 Department of Pediatrics, University Hospital of Split, Spinčićeva 1, 21000 Split, Croatia; 5 Department of Anesthesiology and Intensive Care, University Hospital of Split, Spinčićeva 1, 21000 Split, Croatia; 6 Department of Surgery, School of Medicine, University of Split, Šoltanska 2A, 21000 Split, Croatia; 7 Department of Pediatric Surgery, University Hospital of Split, Spinčićeva 1, 21000 Split, Croatia

**Keywords:** appendicitis, artificial intelligence, machine learning, clinical decision support

## Abstract

Artificial intelligence (AI) and machine learning (ML) are transforming the management of acute appendicitis by enhancing diagnostic accuracy, optimizing treatment strategies, and improving patient outcomes. This study reviews AI applications across all stages of appendicitis care, from triage to postoperative management, using sources from PubMed/MEDLINE, IEEE Xplore, arXiv, Web of Science, and Scopus, covering publications up to 14 February 2025. AI models have demonstrated potential in triage, enabling rapid differentiation of appendicitis from other causes of abdominal pain. In diagnostics, ML algorithms incorporating clinical, laboratory, imaging, and demographic data have improved accuracy and reduced uncertainty. These tools also predict disease severity, aiding decisions between conservative management and surgery. Radiomics further enhances diagnostic precision by analyzing imaging data. Intraoperatively, AI applications are emerging to support real-time decision-making, assess procedural steps, and improve surgical training. Postoperatively, ML models predict complications such as abscess formation and sepsis, facilitating early interventions and personalized recovery plans. This is the first comprehensive review to examine AI’s role across the entire appendicitis treatment process, including triage, diagnosis, severity prediction, intraoperative assistance, and postoperative prognosis. Despite its potential, challenges remain regarding data quality, model interpretability, ethical considerations, and clinical integration. Future efforts should focus on developing end-to-end AI-assisted workflows that enhance diagnosis, treatment, and patient outcomes while ensuring equitable access and clinician oversight.

## 1. Introduction

Machine learning (ML) is increasingly being applied in healthcare to enhance diagnostic accuracy, optimize treatment strategies, and improve patient outcomes [1]. By analyzing medical records, ML models can predict diagnoses, identify high-risk patients, and provide tailored treatment recommendations [2]. Furthermore, ML has demonstrated significant potential in radiological diagnostics, with models capable of detecting tumors, fractures, and other pathological lesions [3]. Beyond diagnostics, ML may also be utilized for developing individualized treatment plans, as well as applications in genomics and genome editing [4,5]. In the field of surgery, the applications of ML and artificial intelligence (AI) extend to a wide array of potential benefits. By analyzing preoperative data, ML models can identify patient-specific risks and assist in surgical planning while offering valuable predictors for postoperative care and possible adverse events. Intraoperatively, AI systems can support decision-making through real-time analysis of procedure steps and progress. These systems integrate electronic medical records with intraoperative video footage, vital signs, instrument tracking, and energy usage data. This comprehensive analysis has the potential to predict and help avoid adverse events during surgery. Additionally, the integration of pre-, intra-, and postoperative data may improve the monitoring of patients during the recovery period, as well as the prediction of postoperative complications. Other AI applications in surgery include automated robotic procedures, real-time decision support, surgical education, and performance evaluation [6,7,8,9,10,11,12,13,14,15,16].

Acute appendicitis (AA) is the most common pathology requiring emergency surgery. Despite its prevalence, diagnosing acute appendicitis continues to present significant challenges. Clinical assessment, while foundational, suffers from low diagnostic accuracy as clinical signs and symptoms have highly variable sensitivity and specificity rates. To further enhance diagnostic accuracy, variable biomarkers can be used. Traditionally, white blood cell count (WBC) and C-reactive protein (CRP) are used independently or as part of diagnostic clinical scores [17]. On the other hand, some others, such as bilirubin, IL-6, ischemia-modified albumin (IMA), calprotectin, leucine-rich α-2-glycoprotein (LRG), or a combination of them (e.g., neutrophile-to-lymphocyte ratio (NLR), APPY1 biomarker panel), are not routinely used in everyday clinical practice [18]. Various scoring systems, including the Alvarado score, Appendicitis Inflammatory Response (AIR) score, Pediatric Appendicitis Score (PAS), and Adult Appendicitis Score (AAS), attempt to standardize diagnosis by combining clinical and laboratory findings, but demonstrate inconsistent sensitivity and specificity across diverse patient populations [19]. Imaging modalities have significantly improved diagnostic accuracy, with ultrasound (US) offering radiation-free assessment, which is particularly valuable in pediatric and pregnant patients. However, it comes with the disadvantage of relying on radiologist’s experience and the potential inability to visualize the appendix during the examination. Computed tomography (CT) provides superior sensitivity and specificity; however, concerns regarding radiation exposure and resource availability temper its universal application. In pregnant patients with suspected AA, magnetic resonance imaging (MRI) emerges as a radiation-free alternative with similar sensitivity and specificity to CT, though accessibility and cost pose barriers to widespread implementation [20].

While ML and AI are beginning to reshape many aspects of surgery, their role in the diagnosis and management of acute appendicitis remains nascent. Several studies have investigated the potential of ML models for diagnosis, severity assessment, treatment planning, and predicting postoperative complications. These models rely on a combination of demographic, clinical, laboratory, radiological, and pathological data.

In this review, we aim to provide a comprehensive overview of the current and potential roles of ML and AI in managing acute appendicitis. Specifically, we will explore their applications in: the triage of patients, diagnosis and severity prediction using clinical findings and imaging techniques, intraoperative decision-making and support, and the prediction of postoperative complications. By addressing these areas, we aim to shed light on the transformative potential of ML and AI in improving the diagnosis, treatment, and outcomes of acute appendicitis.

## 2. Methods

**Figure 1:**
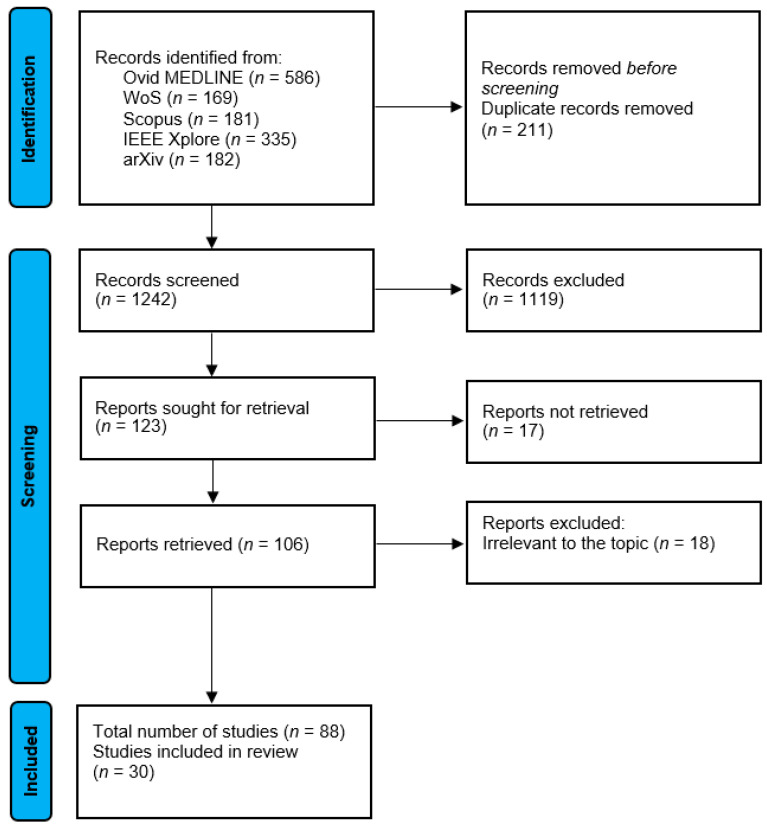
PRISMA flow diagram. MEDLINE—Medical Literature Analysis and Retrieval System Online; WoS—Web of Science; IEEE—Institute of Electrical and Electronics Engineers.

The primary goal of this comprehensive review was to examine how ML and AI models can be used in all possible stages of appendicitis treatment: from triage, through treatment, and follow-up. To ensure a thorough search of the literature and find the relevant studies, I.M. and M.K. conducted searches across multiple databases, including PubMed/MEDLINE, arXiv, IEEE Xplore, Web of Science, and Scopus, covering publications in the English language from their inception to 14 February 2025. The search strategy utilized a combination of Medical Subject Headings (MeSH) terms and keywords related to appendicitis and artificial intelligence in titles or abstracts, incorporating Boolean operators (AND, OR) to refine results. Keywords included terms related to “appendicitis”, “appendectomy”, “artificial intelligence”, “machine learning”, “deep learning”, “neural networks”, “clinical decision support”, “pattern recognition”, and “radiomics”. A review of the titles and abstracts of the articles was then performed. Studies were excluded if the articles were identified as reviews without original data, or if the studies did not primarily focus on appendicitis, or if they were completely unrelated. Studies were included if they utilized ML or AI techniques in any step of appendicitis management (triage, diagnostics, management, intraoperative role, prediction of complications). To maintain objectivity, any differences in opinion or concerns regarding the literature or methodology were thoroughly deliberated among the authors (Figure 1).

## 3. ML and AI Applications in Acute Appendicitis

### 3.1. Role in Triage

AI models designed to improve the triage of patients with acute appendicitis have the potential to enhance both the efficiency and accuracy of initial clinical decision-making. By rapidly analyzing demographic, clinical, and laboratory data, these models could effectively differentiate patients with acute appendicitis from those presenting with abdominal pain due to other causes. This optimized stratification could reduce diagnostic delays, improve the allocation of healthcare resources, and ultimately enhance patient outcomes. To date, several studies have addressed this issue. Singh et al. [21] demonstrated a novel approach to triaging pediatric patients with suspected appendicitis using Machine Learning-Based Medical Directives (MLMDs) at initial assessment. The neural network models achieved impressive performance metrics for abdominal ultrasonography, with a true-positive rate of 0.10, false-positive rate of 0.0006, and positive predictive value of 0.86. The system operates through a dual-pathway approach, where patients either receive immediate test ordering before clinician assessment when the MLMD is activated or follow the standard emergency department workflow if it is not activated. The model evaluates several key clinical indicators, including the presence of abdominal pain, specific mentions of appendicitis concerns in triage notes, and patient vital signs. Implementation demonstrated significant efficiency gains, reducing wait times by an average of 162.7 min per affected patient while maintaining high accuracy. The system includes important safety measures through high positive predictive values to minimize overtesting by the model, with automated ordering only occurring in high-confidence cases, while ensuring that patients not flagged by the system still receive standard care [21].

On a similar note, Su et al. [22] retrospectively analyzed the prediction of AA among emergency department (ED) patients using structured and unstructured free-text data. Among 40,441 patients with appendicitis-related symptoms, 655 (2.3%) adults and 256 (2.2%) children have been confirmed to have AA. The authors used logistic regression (LR) and random forest (RF) models incorporating the natural language processing (NLP) method Doc2Vec to analyze the free-text data. For adult patients, the LR model achieved an area under the curve (AUC) of 0.78 when combining structured and unstructured data, compared to 0.72 for either type alone. For pediatric patients, the combined model reached an AUC of 0.86, versus 0.84 for structured and 0.78 for unstructured data only. Key predictive factors included sex, race, insurance type, triage level, pain severity, and diagnostic services provided. The study demonstrated that incorporating NLP-processed unstructured data improved the models’ predictive accuracy for both adult and pediatric populations [22].

Furthermore, Schipper et al. [23] developed two ML models for the prediction of appendicitis in patients presenting to the ED with acute abdominal pain. The model, with coined name History Intake Vitals Examination (HIVE), used solely ED intake information, vital signs, medical history, and physical examination input. The other model, with the addition of a laboratory test, was called the HIVE-LAB model. The HIVE model achieved an area under the receiver operating characteristic (AUROC) of 0.919, and the HIVE-LAB model achieved an AUROC of 0.923. Both of those models showed high accuracy in predicting appendicitis and outperformed the Alvarado scoring system. In a reader study, three ED physicians with one, five, and ten years of post-qualification experience achieved AUROCs of 0.894, 0.826, and 0.781 without laboratory tests, which increased to 0.923, 0.892, and 0.859 with laboratory test results. Taking their results into account, it is evident that the biggest potential of this model is in predicting AA using triage data without laboratory results [23]. According to the authors, integration of these models could assist ED physicians in early and accurate diagnosis of appendicitis, but we believe that the integration of these models could possibly assist physicians in primary care (family practice) in reducing unnecessary referrals to the ED under the diagnosis of acute appendicitis. As a consequence, there would be fewer unnecessary visits to the EDs, and consequently, overall waiting times could be reduced. Furthermore, the timely diagnosis could be made in primary care so that appendicitis could be treated early, further improving patient outcomes.

### 3.2. Role in Diagnosis and Prediction of Appendicitis Severity

Although diagnosis can sometimes be easily made in patients with typical clinical signs and symptoms, further diagnostic workup is often required, excluding simple laboratory findings. The World Society of Emergency Surgery’s latest recommendations from 2020 for diagnosing and managing appendicitis highlight the need to combine clinical assessment, biochemical markers, and imaging, while factoring in the patient’s age, gender, and underlying health conditions. Furthermore, the recommendations recognize that clinical scores such as the AIR score are valuable tools that can be regularly utilized [20].

ML and deep learning (DL) models have demonstrated promising capabilities in enhancing the diagnostic accuracy of acute appendicitis. DL is a subset of ML that uses multi-layered neural networks for data processing and computations, with the term “deep” referring to the presence of those multiple layers within the algorithm architecture. Multiple algorithms have been employed, including LR, RF, Support Vector Machine (SVM), k-nearest neighbors (KNN), neural networks, XGBoost, decision trees, and more. These models were trained using diverse clinical parameters, such as laboratory findings, demographic data, physical examination results, and imaging features. The models’ development typically involved training on retrospective patient data, with features including, but not limited to, patient history, clinical findings, laboratory results (WBC, neutrophil percentage, CRP), and imaging findings in some cases. The implementation of these AI models has shown potential in reducing diagnostic uncertainty and improving the accuracy of appendicitis diagnosis, with studies reporting performance metrics such as sensitivity, specificity, and AUC as evaluation measures [24].

Apart from potential implications for improved prognosis and better risk stratification, using ML and AI to differentiate between appendicitis subtypes could have therapeutic consequences. Specifically, certain centers perform emergency surgery for complicated cases exclusively, while managing less severe cases with broad-spectrum antibiotics and reserving surgery for those who do not respond to antibiotic treatment. Unlike the classical approach, this strategy considers the non-negligible risks associated with appendicitis surgery. Based on this reasoning, our study group developed ML models for discrimination between complicated appendicitis and either uncomplicated acute appendicitis or no appendicitis. The RF model stands out with 99.7% sensitivity and 17% specificity, which translates to the possibility of saving 14 out of 1000 pediatric patients from undergoing unnecessary surgery. This model outperformed the AIR score in terms of diagnostic accuracy, particularly for distinguishing between complicated and uncomplicated cases [25]. Furthermore, multiple authors have also investigated how ML models can predict the complexity of appendicitis using demographic, clinical, laboratory, and imaging data. For this purpose, most studies relied on histopathological examination of surgical specimens to confirm the classification into complicated and uncomplicated appendicitis, and some used surgical reports. Specifically, gangrenous and/or perforated cases were classified as complicated, while phlegmonous cases were classified as uncomplicated [25,26,27,28,29,30,31,32,33,34]. Among the reviewed studies, the work by Marcinkevics et al. [33] on pediatric patients was one of the most comprehensive, incorporating patient history, laboratory, and imaging data to predict the diagnosis, severity, and management of acute appendicitis. This study achieved areas under the precision–recall curve (AUCPR) of 0.94, 0.70, and 0.92, for diagnosis, severity, and management, respectively [33]. This has led them to the development of a clinical decision support system called the Pediatric Appendicitis Prediction Tool, which is a research prototype but shows how easily these models can be implemented in everyday clinical practice [35]. Similarly, Shikha and Kasem developed a decision tree model called AIPAD to diagnose appendicitis in children aged 0–12. Initial results showed that the model achieved 93.5%  ±  5.8 accuracy, with 91.4% positive predictive value (PPV) and 94.8% negative predictive value (NPV). The prospective validation model performed even better, achieving 97.1% overall accuracy, 96.7% PPV, and 97.4% NPV [36]. Lin et al. [28] demonstrated notable results, achieving an AUC of 0.95 by integrating laboratory findings with CT imaging to predict the type of acute appendicitis. However, despite the model’s high diagnostic accuracy, its reliance on CT imaging raises concerns regarding its clinical applicability, particularly given the imperative to minimize the use of advanced radiological techniques, especially those associated with significant radiation exposure [28]. On the other hand, Akbulut et al. utilized CatBoost algorithms to predict appendicitis types with a high AUC of 0.947, relying solely on laboratory findings and thereby avoiding the need for radiation-based diagnostics [27]. Collectively, the high performance of most of these models suggests that they could be effectively employed in the discrimination of appendicitis types, with potential implications for patient management. Interestingly, Kang et al. [26] used unconventional prognostic variables such as CD4^+^ T cell, helper T cell, B lymphocyte, natural killer (NK) cell counts, and CD4^+^/CD8^+^ ratio to predict pathological types of appendicitis. Their developed LR models, constructed with values of biomarkers, demonstrated robust discriminative capability between simple and purulent appendicitis, achieving an AUC of 0.904 in training and 0.910 in testing sets. With the addition of clinical features, the AUC increased even more to 0.926. In the purulent appendicitis/gangrenous-perforated appendicitis prediction model, the AUC with blood biomarkers was 0.834 for the training and 0.821 for the testing set. When combined with clinical features, the AUC for the testing set increased to 0.854. The model’s performance was further enhanced to an AUC of 0.926 through the incorporation of clinical parameters. In distinguishing purulent from gangrenous-perforated appendicitis, the biomarker-based model yielded AUCs of 0.834 and 0.821 for training and testing sets, respectively. The integration of clinical variables improved the testing set AUC to 0.854 [26]. On a similar note, Reismann et al. applied a supervised ML algorithm to develop a model for differentiation of uncomplicated and complicated appendicitis using gene expression data [37].

However, several issues persist across these models. Most notably, limitations related to the patient selection process and sample size. In terms of patient selection, some models utilize control groups that are artificially constructed, using standardized (validated) statistics and ML techniques [38]. Those methodologies can be problematic for model development, as ideally, models should be based on real patient data to eliminate potential biases [39]. Additionally, some studies had comparative groups with overly broad inclusion criteria, a lack of exclusion criteria apart from suspicion of appendicitis, or undefined inclusion criteria [40,41]. Furthermore, a hallmark characteristic of appendicitis is periumbilical or lower right abdominal quadrant pain [42]. Consequently, including data from patients presenting with epigastric or right upper quadrant pain may introduce bias, as these pain locations, while possible, are highly unlikely. Therefore, careful patient selection is essential. Moreover, appendicitis is an inflammatory disease, and comparing data with healthy patients can also be problematic, as they will not have elevated inflammatory biomarkers such as WBC and CRP, which can inherently create significant statistical differences [32]. Furthermore, some studies have control groups that are diagnosed as having acute appendicitis, but since they were treated conservatively with antibiotics, there was no pathohistological confirmation; thus, the question remains about a possible underlying condition [29,43]. However, a study performed by Marcinkevics et al. stood out with their extensive explanation and reasoning behind that decision [33]. Regarding patient sample size, there is no sample size calculation is necessary in developing AI models; however, they critically rely on large patient datasets, and a lack of sufficient sample size can significantly increase the risk of overfitting [44]. Therefore, some developed models should be tested on larger populations to confirm their results [37,43,45].

Moreover, AI has been utilized in detecting acute appendicitis on ultrasound (US) and MSCT images, a subfield of AI referred to as radiomics. Multiple studies have been published so far, and one of them stands out, which used an 18-layer convolutional neural network (CNN), which is a DL model optimized for processing image-based information and commonly used in visual recognition, image analysis, and segmentation, among other usages. The model in this study was trained on 438 CT scans and then also pretrained on a video collection from the YouTube videos library. This approach significantly improved the performance of the model, with the AUC increasing from 0.724 (95% CI 0.625, 0.823) to 0.810 (95% CI 0.725, 0.895) compared to training the model solely on MSCT images annotated for appendicitis [46]. Marcinkevics et al. [47] expanded on their previously mentioned work in appendicitis diagnostics using ML models by developing interpretable ML models to predict the diagnosis, management, and severity of suspected appendicitis based on ultrasound images. Their approach employed Concept Bottleneck Models (CBMs), which enhance interpretability and allow interaction with high-level concepts that are clinically meaningful. Using this methodology, their model achieved a reported AUROC of 0.80 and AUCPR of 0.92 [47]. On the other hand, Park et al. developed a CNN that was trained for binary classification of appendicitis using data from MSCT, which yielded average sensitivity, specificity, and accuracy of 90.2%, 92%, and 91.5%, respectively [48]. Similarly, Liang et al. [49] developed a combined DL and radiomics model to differentiate between uncomplicated and complicated appendicitis with external validation cohorts from three different centers. In the total validation cohort, their model achieved an AUC of 0.799 and surpassed the conventional combined model, the DL radiomics model, and the radiologist’s visual diagnosis [49]. Furthermore, Zhao et al. [50] investigated the use of clinical information combined with radiomics models to differentiate between simple and complicated appendicitis on CT images. They developed three models: a CT based model, which used clinical data (age, gender, body temperature, laboratory data) combined with CT features; a radiomics model; and a combined model, which integrated clinical data with the radiomics model with the combined model, showing the best performance with an average AUC of 0.817. This study demonstrated that integrating radiomics features with clinical information could provide a more accurate approach to distinguishing between simple and complicated appendicitis, potentially helping clinicians make even more informed treatment decisions [50].

### 3.3. Intraoperative Role

The integration of ML and AI models into the intraoperative management of appendicitis holds transformative potential for enhancing surgical precision and safety. These models can potentially assist in real-time analysis of intraoperative findings by grading the complexity of each case, ensuring tailored decision-making for surgeons. Additionally, these systems could detect and evaluate the completion of each procedural step, helping maintain adherence to safety protocols. Beyond improving intraoperative assessments, AI offers the potential for direct communication with senior surgeons through video or alarms when procedures exceed expected timeframes, ensuring timely interventions. Finally, AI could be a valuable tool for training surgical residents by providing objective feedback on procedural performance and adherence to protocols [6,7,8,9,10,11,12,13,15].

However, data on the role of AI in the intraoperative management of appendicitis remains limited, with only one study addressing the topic. Dayan et al. conducted a validation and reliability study on the implementation of AI-based models using computer vision in laparoscopic appendectomy [51]. Senior surgeons agreed that the case complexity assessed by AI was accurate in 76.9% to 94.4% of cases. Additionally, senior surgeons rated trainee adherence to safety protocols as high, with the complete Critical View of Safety (CVS) being achieved in 92% and 91.4% of cases, according to their evaluations. The AI model accurately assessed the completeness of the CVS in 84.4% of cases. However, when the AI model determined that the CVS was complete, surgeons agreed with this assessment in 99.8% and 96% of cases. The authors note that the model appears to be less accurate in assessing partially completed CVS, attributing this to the model’s design, which evaluates CVS at the moment before the appendix excision and does not account for earlier phases of CVS completion. Given the potential of ML to optimize outcomes and support surgeons during laparoscopic appendectomies, further development and validation of these models are essential. With the limited amount of published data on this topic, additional research is needed to refine these tools and explore their use in training surgical residents.

### 3.4. Prediction of Postoperative Complications and Prognosis

There are several well-recognized postoperative complications of acute appendicitis, the most notable being intestinal obstruction, surgical site infection, intra-abdominal abscess formation, and sepsis [52]. Although such complications are uncommon (especially sepsis), AI-based models may offer valuable support in predicting their occurrence, thereby opening new avenues for preventive strategies. Nonetheless, only a limited number of studies have addressed this issue to date. A study by Alramadhan et al. [53] involved 1574 patients and demonstrated that two artificial neural networks (ANNs) with distinct architectures effectively predicted abscess formation following appendectomy. This predictive performance was based on a range of variables, including postoperative WBCs, intraoperative diagnoses, surgical duration, completion of antibiotic therapy, body temperature at imaging time points, and patient weight. In the first model (sensitivity 70%, specificity 93.6%), the five most highly weighted variables were postoperative WBCs, the surgeon’s final intraoperative diagnosis, completion of antibiotic therapy, body weight, and surgical duration. In contrast, the second model (sensitivity 82%, specificity 84.6%) identified postoperative temperature (recorded at the time of imaging) as the most influential variable, while the remaining four variables were consistent with those of the first model. Yet, it has to be noted that ANNs are inherently prone to data overfitting, which can lead to potentially misleading results. A separate small-scale study (*n* = 163) aimed to predict postoperative appendicitis complications based on various demographic, clinical, and surgical data [54]. The model predicted the need for intensive care and an intensive care unit stay longer than 24 h with satisfactory accuracy (77% and 88%, respectively). However, the small sample size casts doubt on the wider applicability of these results, especially since the incidence of relevant postoperative complications is not very high in most centers. Bunn et al. aimed to establish if sepsis and septic shock post appendectomy might be predicted by demographic, clinical, and surgical data on a sample of over 220,000 appendectomies [55]. The model with the highest accuracy was constructed using an ensemble of the best models, including LR, RF, and XGB (AUC 0.71; 95% CI, 0.69–0.73). Variables with the greatest influence on the model were heart failure diagnosis or recent exacerbation, transfusion of more than one pack of red blood cells within 72 h after surgery, and preoperative acute renal failure. Finally, a unique contribution to the field was made by Ghomrawi et al. [56], who utilized Fitbit smart watches for the postoperative monitoring of pediatric patients following appendectomy. The device effectively tracked multimodal data about daily physical activity, heart rate, and sleep, in detecting abnormalities in the early postoperative period of children recovering from appendectomy. By integrating these data with clinical and demographic variables, the authors developed a machine learning model (a balanced random forest classifier) that achieved an accuracy of 83% in identifying abnormal recovery days in cases of complicated appendicitis and 70% in uncomplicated appendicitis [56].

### 3.5. Summary of the Relevant Publications Based on AI Task Type

Table 1 summarizes the relevant publications based on AI task type that were referenced in several categories: Triage and early diagnosis, diagnosis and severity prediction, radiological imaging, intraoperative assistance, postoperative complications and prognosis, and histopathological analysis [21,22,23,24,25,26,27,28,33,46,47,48,49,50,51,53,54,55,56,57].

## 4. Future Directions and Limitations

**Figure 2:**
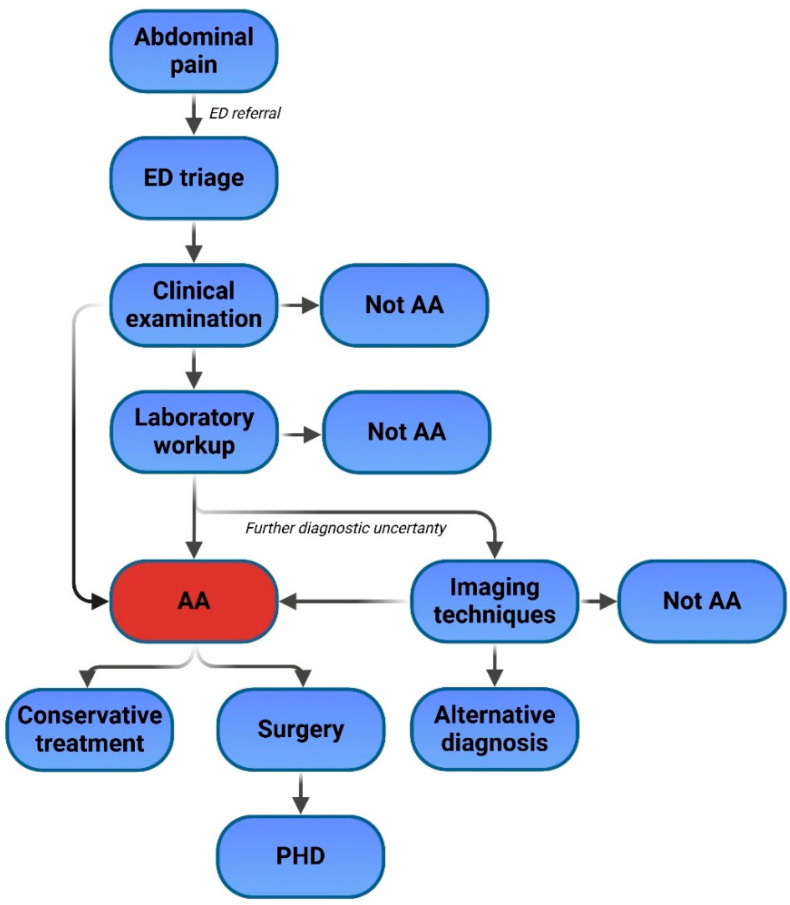
Current state of the diagnostic process of acute appendicitis. AA—acute appendicitis, ED—emergency department, PHD—pathohistological diagnosis.

Artificial intelligence holds the potential to revolutionize the diagnostic and treatment processes for acute appendicitis by enhancing diagnostic accuracy, speed, and consistency. Future directions in AI development should focus on integrating these technologies into various stages of patient care to maximize their impact. In the diagnostic processes, AI models could evolve to include multi-modal data integration, combining clinical, laboratory, radiological, and demographic data to create highly accurate, real-time diagnostic tools. These tools could improve triage by identifying high-risk populations, streamlining diagnostic workflows, and reducing waiting times in emergency departments. As a next step, AI models could analyze demographic and anamnestic data, laboratory results, and clinical findings to stratify patients into risk categories or directly diagnose acute appendicitis. When diagnostic uncertainty persists, AI systems utilizing radiomics could analyze radiological imaging or provide accurate diagnostic insights (Figure 2).

Furthermore, AI models could be used to predict the severity of appendicitis to decide on the best treatment options, antibiotic treatment, or to assist in deciding between conservative and surgical treatment. With further development and widespread usage of digital pathology, AI models could also potentially analyze histological specimens autonomously, providing consistent and precise diagnoses. Collectively, these advancements could standardize and streamline the diagnostic process, reducing variability among clinicians and ensuring equitable care.

**Figure 3:**
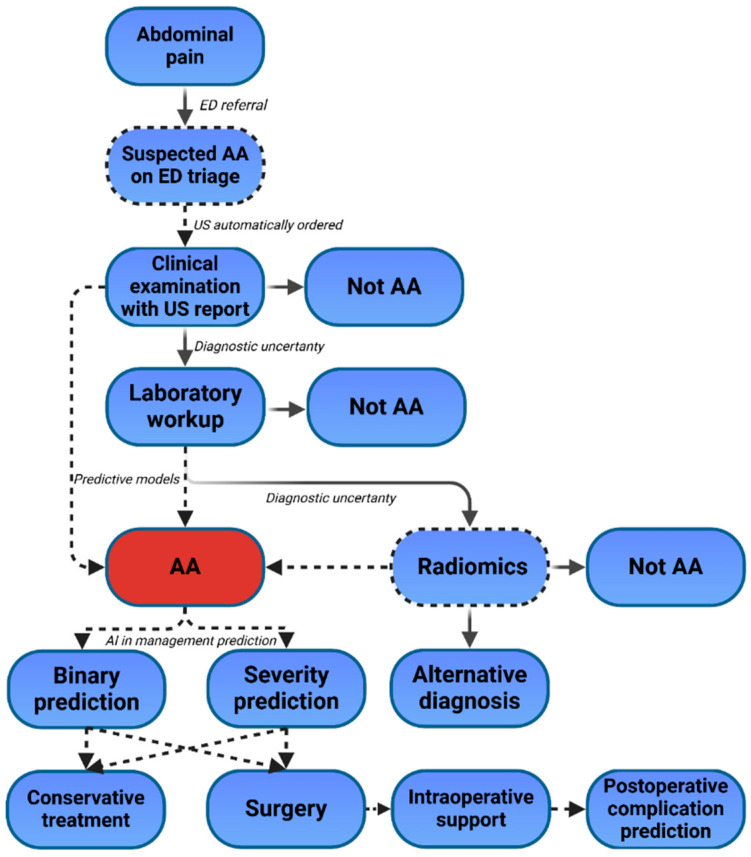
Potential future AI-assisted workflow for acute appendicitis treatment process. Dotted lines and boxes show potential AI-assisted pathways or points in the suggested workflow. AA—acute appendicitis, ED—emergency department, US—ultrasound.

In the surgical domain, AI has the potential to reshape both training and operative procedures. AI-powered tools can serve as educational platforms for surgeons, offering automated performance assessments and feedback based on video analysis of procedures. By evaluating intraoperative benchmarks, such as the completion of the critical view of safety, AI can objectively assess surgical competency and guide targeted skill development. During surgery, real-time AI-driven guidance systems could support surgeons by identifying anatomical structures, predicting surgery duration, or providing alerts for deviations from standard protocols. These capabilities could reduce intraoperative errors, enhance patient safety, and improve surgical outcomes. Postoperatively, AI models could play a critical role in predicting and mitigating complications. By continuously analyzing patient recovery data, such as vitals, laboratory values, and imaging, AI systems could provide early warning of complications, allowing for timely interventions. Additionally, AI could facilitate personalized recovery plans by predicting patient-specific risks and tailoring follow-up care. Based on the above-mentioned reasoning, we designed an integrated AI-assisted workflow for putative patients presenting with symptoms of acute appendicitis (Figure 3).

The comprehensive integration of AI in appendicitis management has demonstrated remarkable potential across multiple clinical domains. However, a critical component in the diagnostic chain—histopathological analysis—remains relatively underexplored in terms of AI implementation. The emergence of digital pathology, coupled with advanced whole-slide imaging capabilities, presents a unique opportunity to incorporate AI-driven solutions into the microscopic evaluation of appendiceal specimens [57]. AI models could be developed and validated to distinguish between normal appendiceal architecture and various inflammatory states, potentially extending their capability to identify specific pathological entities, including acute suppurative inflammation, gangrenous changes, and neoplastic transformations. Furthermore, the development of these models could potentially improve diagnostic precision, standardize pathological reporting procedures, and minimize inter-observer variability. Moreover, the implementation of AI solutions in digital pathology could accelerate specimen analysis, facilitating more rapid clinical decision-making and potentially leading to improved patient outcomes.

The incorporation of AI-powered pathological analysis would complete a potential end-to-end, AI-augmented clinical pathway for AA, encompassing the following stages:

- Emergency department (ED) triage optimization
- Initial diagnostic support during clinical evaluation
- Automated radiological interpretation through radiomics
- Treatment strategy optimization (conservative versus surgical approach)
- Computer-assisted intraoperative guidance
- Predictive analytics for postoperative complications and prognosis
- Automated histopathological analysis through digital pathology platforms

Such comprehensive integration of AI technologies represents a significant advancement toward establishing a fully digitalized and intelligent healthcare ecosystem for AA management, while preserving the essential role of healthcare professionals in supervising and validating AI-generated insights. This synergistic relationship between AI systems and medical expertise could potentially revolutionize the standard of care in AA management.

The continued evolution of AI, particularly the integration of sophisticated natural language processing models exemplified by ChatGPT (version 4), suggests a potential paradigm shift in healthcare delivery, where physician involvement could be reduced mainly to surgical interventions, while other aspects of patient care and treatment, such as making diagnoses and choosing treatment options, could be automated through AI systems [58].

Despite these promising opportunities, several limitations must be addressed. First, many current studies are based on retrospective data with heterogeneous methodologies, limiting the ability to generalize findings to broader clinical settings. Consequently, there is a need for more prospective multicentric studies to improve the generalizability of findings. Such studies would enable the validation of AI models on diverse datasets, ensuring their reliability and applicability across diverse patient populations and clinical settings.

Second, data quality and standardization issues remain prevalent, making it difficult to develop robust and comparable AI models across institutions. Additionally, future research should also explore the ethical and practical implications of widespread AI adoption in healthcare. Efforts must be made to ensure these models are transparent, interpretable, and validated across diverse populations to avoid biases and disparities. This approach is crucial for building trust in AI systems and minimizing the risk of bias or inaccuracies when applied in real-world scenarios.

Furthermore, the integration of AI models into existing hospital computer systems could present a significant challenge. We believe that many of the hospital systems are based on legacy infrastructure, which could pose a major challenge in seamless AI implementation. Upgrading or developing new systems to accommodate an AI solution will demand substantial financial investment and technical expertise. Moreover, ensuring compatibility between AI tools and electronic health records would be complex, as data formats and standards in recording data differ between institutions.

Lastly, legal and regulatory issues further complicate the adoption of AI in healthcare. Compliance with regulations, such as the General Data Protection Regulation (GDPR) in Europe, is essential to protect patients’ privacy and ensure ethical data usage. As GDPR mandates strict guidelines on data collection, storage, and sharing, it can pose a barrier to training AI models that require large-scale datasets. Furthermore, as AI tools rely on processing vast amounts of information, the secure and efficient storage of data is crucial to prevent unwanted access to such data. Beyond that, a specific regulatory framework should be created so there is no uncertainty over liability in cases where AI-generated recommendations or actions lead to adverse outcomes. Ethical considerations further amplify these challenges. The use of AI tools in clinical decision-making raises critical questions about both accountability and trust. If an AI tool produced an incorrect diagnosis or treatment recommendation, it remains unclear whether responsibility lies with the creators of the tools, the institution that implemented it, or the clinician who used and applied it. This can undermine trust between clinicians and patients. Moreover, there is a risk of overreliance on AI tools and their outputs by clinicians who may fail to critically evaluate their accuracy. Ensuring that clinicians remain central in decision-making processes is, therefore, essential to preventing such risks. Lastly, for these systems to be used as medical tools, obtaining regulatory approval from bodies such as the FDA or EMA is critical. Such approval ensures that the models meet strict standards for clinical use, addressing concerns related to accuracy, transparency, and patient safety while fostering trust among healthcare providers and patients. The FDA’s traditional premarket approval pathway was not designed for algorithms that evolve over time, necessitating the development of novel regulatory frameworks. The FDA has published marketing submission recommendations for a predetermined change control plan for artificial intelligence-enabled device software functions. This guidance outlines a regulatory framework for AI-enabled medical devices through Predetermined Change Control Plans (PPCPs), which, assuming all prerequisites are met, allow manufacturers to implement certain modifications without additional premarket submissions, as the FDA has recognized that AI development can be an iterative process to improve models. That way, developers have some guidelines in developing software compliant with possible future regulations [59].

To address these challenges effectively, collaboration among engineers, data scientists, clinicians, healthcare administrators, and policymakers is essential. This multidisciplinary approach can help overcome barriers related to data sharing, privacy concerns, implementation logistics, and proper interpretation of AI outputs. By addressing these issues proactively, the healthcare sector can harness the potential of AI while safeguarding ethical principles and ensuring equitable access for all patients. We believe that future studies should implement a prospective design, have standardized data collection protocols, and implement a practice of collaboration with other centers so that multicenter data can be used in developing models and validation sets to improve the generalizability of already developed models, as well as those in the development phase.

## 5. Conclusions

Recent advances in the field of artificial intelligence (AI) are reshaping appendicitis management by moving beyond traditional diagnostic and treatment approaches. From optimizing emergency department triage to enhancing postoperative recovery, AI-driven tools can significantly improve diagnostic accuracy, workflow efficiency, and ensure equitable care delivery. In addition, radiomics and deep learning models enhance diagnostic imaging, improving the detection of appendicitis. Intraoperatively, AI-assisted computer vision could provide real-time guidance, procedural assessment, and risk prediction to enhance surgical precision and outcomes. Postoperatively, predictive analytics can help predict complications early and tailor follow-up care to individual patient needs.

The future of AI in appendicitis management lies in the development of fully integrated workflows that seamlessly combine emergency triage, diagnostic assessment, intraoperative guidance, postoperative monitoring, and digital pathology into a cohesive system. While many AI-driven modalities, such as radiomics and predictive analytics, have already made significant advancements, digital pathology for appendicitis remains entirely unexplored, while intraoperative AI applications are still in their early stages. These areas represent critical opportunities for future development. However, several challenges must be addressed, including ethical considerations, privacy regulations, model interpretability, and clinical integration. Addressing these issues through multidisciplinary collaboration and rigorous validation studies will be key to realizing the full potential of AI in appendicitis treatment.

In conclusion, AI has the potential to revolutionize the management of acute appendicitis by integrating seamlessly across all stages of care, from triage and diagnosis to surgical treatment and postoperative recovery, all the way to the specimen analysis. This comprehensive approach means that AI could be effectively employed from the patient’s first step into the emergency department to their final step out of the hospital, fundamentally transforming the quality and consistency of care.

## Figures and Tables

**Table 1:** Categorization of some of the referenced papers based on task types.

Study	Study Design and Dataset	AI Model(s) Used	Data Used	Clinical Implications
AI in triage and early diagnosis				
Singh et al. [21]	Retrospective study on data from 77,219 pediatric patients	Various ML models	Clinical data	Optimized early decision-making and reducing triage wait times
Su et al. [22]	Retrospective study on data from 40,441 ED patients	LR, RF, NLP-Doc2Vec	Clinical data	Enhanced triage efficiency
Schipper et al. [23]	Retrospective study on data from 336 ED patients	HIVE and HIVE-LAB models	Clinical data and standard laboratory tests	Early and accurate appendicitis prediction outperforming Alvarado score
AI in diagnosis and severity prediction				
Issaiy et al. [24]	Systematic review of AI diagnostic models	Various ML models	/	AI can aid in diagnostics and in risk stratification for appendicitis severity
Males et al. [25]	Retrospective study on data from 551 pediatric patients	LR, RF, XGBoost	Clinical data, standard laboratory tests, and AIR score	Reducing negative appendectomy rates
Kang et al. [26]	Retrospective study on data from 136 patients	LR	Clinical data, standard and unconventional biomarkers	Predicting appendicitis severity
Akbulut et al. [27]	Retrospective study on data from 1797 patients	CatBoost	Clinical data, standard laboratory tests	Predicting appendicitis severity
Lin et al. [28]	Retrospective study on 441 patients	ANNs	Clinical data, standard laboratory tests, and MSCT findings	AI-integrated imaging enhances diagnostic accuracy
Marcinkevics et al. [33]	Retrospective study on 430 pediatric patients	LR, RF, GBM	Clinical data, standard laboratory tests, US findings, PAS	Using ML in diagnostics, management, and severity prediction, an online decision support tool
AI in radiological imaging				
Rajpurkar et al. [46]	Retrospective study on 646 CT scans	CNN	Images from CT scans and YouTube videos	Automated detection of appendicitis on CT scans
Marcinkevics et al. [47]	Retrospective study on 579 pediatric patients	MVCBM, SSMBCBM	Clinical data, standard laboratory tests, AS and PAS, US images	Using CBMs for predicting diagnosis, management, and severity, leveraging US images
Park et al. [48]	Retrospective study on 667 CT scans with external validation	CNN	Images from CT scans	Automated detection of appendicitis on CT scans
Liang et al. [49]	Retrospective multicenter study on 1165 CT scans	Combined model, DL radiomics	Images from CT scans	Differentiation of complicated and uncomplicated appendicitis
Zhao et al. [50]	Retrospective study on 334 patients	Radiomics and combined models	Clinical data, standard laboratory tests, and images from CT scans	Integrating clinical data and laboratory tests with a radiomics model to differentiate between simple and complicated appendicitis
AI in intraoperative assistance				
Dayan et al. [51]	Retrospective study on 499 appendectomy videos	Commercial computer vision AI Model	Automated annotations	AI-assisted guidance for laparoscopic appendectomy, predicting operative time and intraoperative course
AI in postoperative complications and prognosis				
Alramadhan et al. [53]	Retrospective study on 1574 patients	ANNs	Clinical data, standard laboratory tests, intraoperative data	Predicting postoperative intra-abdominal abscess
Eickhoff et al. [54]	Retrospective study on 163 patients	RF	Clinical data, standard laboratory tests, intraoperative data	Postoperative care planning
Bunn et al. [55]	Retrospective study on 223,214 patients, data from the ACS NSQIP database	LR, SVM, RF, XGBoost	Clinical data, standard laboratory tests	Recognizing patients at risk of postoperative sepsis
Ghomrawi et al. [56]	Prospective study on 162 pediatric patients	Balanced RF	Data acquired from a wearable device	Detecting abnormal recovery symptoms and complications up to two days before occurring
AI in histopathological analysis				
McGenity et al. [57]	Systematic review of AI in digital pathology	Various AI pathology models	/	Need for developing a tool to identify appendicitis in appendix specimens

ACS NSQIP—American College of Surgeons National Surgical Quality Improvement Program; AI—Artificial Intelligence; AIR score—Appendicitis Inflammatory Response score; ANNs—Artificial Neural Networks; CBMs—Cluster-Based Models; CNN—Convolutional Neural Network; DL—Deep Learning; Doc2Vec—Document to Vector; HIVE—High-Value Extractor; LR—Logistic Regression; ML—Machine Learning; MSCT—Multi-Slice Computed Tomography; MVCBM—Model-Based Clustering with Bayesian Model Averaging; NLP—Natural Language Processing; PAS—Pediatric Appendicitis Score; RF—Random Forest; SSMBCBM—Semi-Supervised Model-Based Clustering with Bayesian Model Averaging; SVM—Support Vector Machine; US—Ultrasound; XGBoost—Extreme Gradient Boosting.

## Data Availability

Not applicable.

## References

[1] Habehh H., Gohel S. (2021). Machine Learning in Healthcare. Curr. Genom..

[2] Li Y.H., Li Y.L., Wei M.Y., Li G.Y. (2024). Innovation and challenges of artificial intelligence technology in personalized healthcare. Sci. Rep..

[3] Li M., Jiang Y., Zhang Y., Zhu H. (2023). Medical image analysis using deep learning algorithms. Front. Public. Health.

[4] Gallo C. (2023). Artificial Intelligence for Personalized Genetics and New Drug Development: Benefits and Cautions. Bioengineering.

[5] Dixit S., Kumar A., Srinivasan K., Vincent P., Ramu Krishnan N. (2023). Advancing genome editing with artificial intelligence: Opportunities, challenges, and future directions. Front. Bioeng. Biotechnol..

[6] Mahajan A., Esper S., Oo T.H., McKibben J., Garver M., Artman J., Klahre C., Ryan J., Sadhasivam S., Holder-Murray J. (2023). Development and Validation of a Machine Learning Model to Identify Patients Before Surgery at High Risk for Postoperative Adverse Events. JAMA Netw. Open.

[7] Bellini V., Valente M., Turetti M., Del Rio P., Saturno F., Maffezzoni M., Bignami E. (2022). Current Applications of Artificial Intelligence in Bariatric Surgery. Obes. Surg..

[8] Gumbs A.A., Frigerio I., Spolverato G., Croner R., Illanes A., Chouillard E., Elyan E. (2021). Artificial Intelligence Surgery: How Do We Get to Autonomous Actions in Surgery?. Sensors.

[9] Humm G., Harries R.L., Stoyanov D., Lovat L.B. (2021). Supporting laparoscopic general surgery training with digital technology: The United Kingdom and Ireland paradigm. BMC Surg..

[10] Lin C.C., Chen Y.P., Chiang C.C., Chang M.C., Lee O.K. (2020). Real-Time Streaming of Surgery Performance and Intraoperative Imaging Data in the Hybrid Operating Room: Development and Usability Study. JMIR Med. Inform..

[11] Müller L.R., Petersen J., Yamlahi A., Wise P., Adler T.J., Seitel A., Kowalewski K.-F., Müller B., Kenngott H., Nickel F. (2022). Robust hand tracking for surgical telestration. Int. J. Comput. Assist. Radiol. Surg..

[12] Hashimoto D.A., Rosman G., Witkowski E.R., Stafford C., Navarette-Welton A.J., Rattner D.W., Lillemoe K.D., Rus D.L., Meireles O.R. (2019). Computer Vision Analysis of Intraoperative Video: Automated Recognition of Operative Steps in Laparoscopic Sleeve Gastrectomy. Ann. Surg..

[13] Liu Y., Zhao Z., Chang F., Hu S. (2020). An Anchor-Free Convolutional Neural Network for Real-Time Surgical Tool Detection in Robot-Assisted Surgery. IEEE Access.

[14] Saeidi H., Opfermann J.D., Kam M., Wei S., Leonard S., Hsieh M.H., Kang J.U., Krieger A. (2022). Autonomous robotic laparoscopic surgery for intestinal anastomosis. Sci. Robot..

[15] Kawamura M., Endo Y., Fujinaga A., Orimoto H., Amano S., Kawasaki T., Kawano Y., Masuda T., Hirashita T., Kimura M. (2023). Development of an artificial intelligence system for real-time intraoperative assessment of the Critical View of Safety in laparoscopic cholecystectomy. Surg. Endosc..

[16] Loftus T.J., Tighe P.J., Filiberto A.C., Efron P.A., Brakenridge S.C., Mohr A.M., Rashidi P., Upchurch G.R., Bihorac A. (2020). Artificial Intelligence and Surgical Decision-making. JAMA Surg..

[17] Skjold-Ødegaard B., Søreide K. (2022). The diagnostic differentiation challenge in acute appendicitis: How to distinguish between uncomplicated and complicated appendicitis in adults. Diagnostics.

[18] Tintor G., Jukić M., Šupe-Domić D., Jerončić A., Pogorelić Z. (2023). Diagnostic Utility of Serum Leucine-Rich α-2-Glycoprotein 1 for Acute Appendicitis in Children. J. Clin. Med..

[19] Moris D., Paulson E.K., Pappas T.N. (2021). Diagnosis and management of acute appendicitis in adults: A review. JAMA.

[20] Di Saverio S., Podda M., De Simone B., Ceresoli M., Augustin G., Gori A., Rashidi P., Upchurch G.R., Bihorac A. (2020). Diagnosis and treatment of acute appendicitis: 2020 update of the WSES Jerusalem guidelines. World J. Emerg. Surg..

[21] Singh D., Nagaraj S., Mashouri P., Drysdale E., Fischer J., Goldenberg A., Brudno M. (2022). Assessment of Machine Learning–Based Medical Directives to Expedite Care in Pediatric Emergency Medicine. JAMA Netw. Open.

[22] Su D., Li Q., Zhang T., Veliz P., Chen Y., He K., Mahajan P., Zhang X. (2022). Prediction of acute appendicitis among patients with undifferentiated abdominal pain at emergency department. BMC Med. Res. Methodol..

[23] Schipper A., Belgers P., O’Connor R., Jie K.E., Dooijes R., Bosma J.S., Kurstjens S., Kusters R., van Ginneken B., Rutten M. (2024). Machine-learning based prediction of appendicitis for patients presenting with acute abdominal pain at the emergency department. World J. Emerg. Surg..

[24] Issaiy M., Zarei D., Saghazadeh A. (2023). Artificial Intelligence and Acute Appendicitis: A Systematic Review of Diagnostic and Prognostic Models. World J. Emerg. Surg..

[25] Males I., Boban Z., Kumric M., Vrdoljak J., Berkovic K., Pogorelic Z., Bozic J. (2024). Applying an explainable machine learning model might reduce the number of negative appendectomies in pediatric patients with a high probability of acute appendicitis. Sci. Rep..

[26] Kang C.B., Li X.W., Hou S.Y., Chi X.Q., Shan H.F., Zhang Q.J., Liu T.-J. (2021). Preoperatively predicting the pathological types of acute appendicitis using machine learning based on peripheral blood biomarkers and clinical features: A retrospective study. Ann. Transl. Med..

[27] Akbulut S., Yagin F.H., Cicek I.B., Koc C., Colak C., Yilmaz S. (2023). Prediction of Perforated and Nonperforated Acute Appendicitis Using Machine Learning-Based Explainable Artificial Intelligence. Diagnostics.

[28] Lin H.A., Lin L.T., Lin S.F. (2023). Application of Artificial Neural Network Models to Differentiate Between Complicated and Uncomplicated Acute Appendicitis. J. Med. Syst..

[29] Phan-Mai T.A., Thai T.T., Mai T.Q., Vu K.A., Mai C.C., Nguyen D.A. (2023). Validity of Machine Learning in Detecting Complicated Appendicitis in a Resource-Limited Setting: Findings from Vietnam. Biomed. Res. Int..

[30] Li P., Zhang Z., Weng S., Nie H. (2023). Establishment of predictive models for acute complicated appendicitis during pregnancy—A retrospective case-control study. Int. J. Gynaecol. Obstet..

[31] Xia J., Wang Z., Yang D., Li R., Liang G., Chen H., Heidari A.A., Turabieh H., Mafarja M., Pan Z. (2022). Performance optimization of support vector machine with oppositional grasshopper optimization for acute appendicitis diagnosis. Comput. Biol. Med..

[32] Aydin E., Türkmen İ U., Namli G., Öztürk Ç., Esen A.B., Eray Y.N., Eroğlu E., Akova F. (2020). A novel and simple machine learning algorithm for preoperative diagnosis of acute appendicitis in children. Pediatr. Surg. Int..

[33] Marcinkevics R., Reis Wolfertstetter P., Wellmann S., Knorr C., Vogt J.E. (2021). Using Machine Learning to Predict the Diagnosis, Management and Severity of Pediatric Appendicitis. Front. Pediatr..

[34] Reismann J., Romualdi A., Kiss N., Minderjahn M.I., Kallarackal J., Schad M., Reismann M. (2019). Diagnosis and classification of pediatric acute appendicitis by artificial intelligence methods: An investigator-independent approach. PLoS ONE.

[35] Medical Data Science Research Group, ETH Zurich Pediatric Appendicitis Prediction Tool. https://papt.inf.ethz.ch.

[36] Shikha A., Kasem A. (2022). The Development and Validation of Artificial Intelligence Pediatric Appendicitis Decision-Tree for Children 0 to 12 Years Old. Eur. J. Pediatr. Surg..

[37] Reismann J., Kiss N., Reismann M. (2021). The application of artificial intelligence methods to gene expression data for differentiation of uncomplicated and complicated appendicitis in children and adolescents—a proof of concept study. BMC Pediatr..

[38] Ghareeb W.M., Draz E., Chen X., Zhang J., Tu P., Madbouly K., Moratal M., Ghanem A., Amer M., Hassan A. (2024). Multicenter validation of an artificial intelligence (AI)—based platform for the diagnosis of acute appendicitis. Surgery.

[39] Giuffrè M., Shung D.L. (2023). Harnessing the power of synthetic data in healthcare: Innovation, application, and privacy. npj Digit. Med..

[40] Akmese O.F., Dogan G., Kor H., Erbay H., Demir E. (2020). The Use of Machine Learning Approaches for the Diagnosis of Acute Appendicitis. Emerg. Med. Int..

[41] Akgül F., Er A., Ulusoy E., Çağlar A., Çitlenbik H., Keskinoğlu P., Şişman A.R., Karakuş O.Z., Özer E., Duman M. (2021). Integration of Physical Examination, Old and New Biomarkers, and Ultrasonography by Using Neural Networks for Pediatric Appendicitis. Pediatr. Emerg. Care.

[42] Pogorelić Z., Janković Marendić I., Čohadžić T., Jukić M. (2023). Clinical Outcomes of Daytime Versus Nighttime Laparoscopic Appendectomy in Children. Children.

[43] Hsieh C.-H., Lu R.-H., Lee N.-H., Chiu W.-T., Hsu M.-H., Li Y.-C. (2011). Novel solutions for an old disease: Diagnosis of acute appendicitis with random forest, support vector machines, and artificial neural networks. Surgery.

[44] Zantvoort K., Nacke B., Görlich D., Hornstein S., Jacobi C., Funk B. (2024). Estimation of minimal data sets sizes for machine learning predictions in digital mental health interventions. npj Digit. Med..

[45] Prabhudesai S.G., Gould S., Rekhraj S., Tekkis P.P., Glazer G., Ziprin P. (2008). Artificial neural networks: Useful aid in diagnosing acute appendicitis. World J. Surg..

[46] Rajpurkar P., Park A., Irvin J., Chute C., Bereket M., Mastrodicasa D., Langlotz C.P., Lungren M.P., Ng A.Y., Patel B.N. (2020). AppendiXNet: Deep Learning for Diagnosis of Appendicitis from A Small Dataset of CT Exams Using Video Pretraining. Sci. Rep..

[47] Marcinkevičs R., Reis Wolfertstetter P., Klimiene U., Chin-Cheong K., Paschke A., Zerres J., Denzinger M., Niederberger D., Wellmann S., Ozkan E. (2024). Interpretable and intervenable ultrasonography-based machine learning models for pediatric appendicitis. Med. Image Anal..

[48] Park J.J., Kim K.A., Nam Y., Choi M.H., Choi S.Y., Rhie J. (2020). Convolutional-neural-network-based diagnosis of appendicitis via CT scans in patients with acute abdominal pain presenting in the emergency department. Sci. Rep..

[49] Liang D., Fan Y., Zeng Y., Zhou H., Zhou H., Li G., Liang Y., Zhong Z., Chen D., Chen A. (2024). Development and Validation of a Deep Learning and Radiomics Combined Model for Differentiating Complicated From Uncomplicated Acute Appendicitis. Acad. Radiol..

[50] Zhao Y., Wang X., Zhang Y., Liu T., Zuo S., Sun L., Zhang J., Wang K., Liu J. (2024). Combination of clinical information and radiomics models for the differentiation of acute simple appendicitis and non simple appendicitis on CT images. Sci. Rep..

[51] Dayan D., Dvir N., Agbariya H., Nizri E. (2024). Implementation of artificial intelligence-based computer vision model in laparoscopic appendectomy: Validation, reliability, and clinical correlation. Surg. Endosc..

[52] Wu M.-C., Tsou H.-K., Lin C.-L., Wei J.C.-C. (2020). Incidence and risk of sepsis following appendectomy: A nationwide population-based cohort study. Sci. Rep..

[53] Alramadhan M.M., Al Khatib H.S., Murphy J.R., Tsao K., Chang M.L. (2022). Using Artificial Neural Networks to Predict Intra-Abdominal Abscess Risk Post-Appendectomy. Ann. Surg. Open.

[54] Eickhoff R.M., Bulla A., Eickhoff S.B., Heise D., Helmedag M., Kroh A., Schmitz S.M., Klink C.D., Neumann U.P., Lambertz A. (2022). Machine learning prediction model for postoperative outcome after perforated appendicitis. Langenbecks Arch. Surg..

[55] Bunn C., Kulshrestha S., Boyda J., Balasubramanian N., Birch S., Karabayir I., Baker M., Luchette F., Modave F., Akbilgic O. (2021). Application of machine learning to the prediction of postoperative sepsis after appendectomy. Surgery.

[56] Ghomrawi H.M.K., O’Brien M.K., Carter M., Macaluso R., Khazanchi R., Fanton M., DeBoer C., Linton S.C., Zeineddin S., Pitt J.B. (2023). Applying machine learning to consumer wearable data for the early detection of complications after pediatric appendectomy. NPJ Digit. Med..

[57] McGenity C., Clarke E.L., Jennings C., Matthews G., Cartlidge C., Freduah-Agyemang H., Stocken D.D., Treanor D. (2024). Artificial intelligence in digital pathology: A systematic review and meta-analysis of diagnostic test accuracy. npj Digit. Med..

[58] Sanduleanu S., Ersahin K., Bremm J., Talibova N., Damer T., Erdogan M., Kottlors J., Goertz L., Bruns C., Maintz D. (2024). Feasibility of GPT-3.5 versus Machine Learning for Automated Surgical Decision-Making Determination: A Multicenter Study on Suspected Appendicitis. AI.

[59] US Food and Drug Administration (2024). Marketing Submission Recommendations for a Predetermined Change Control Plan for Artificial Intelligence-Enabled Device Software Functions: Guidance for Industry and Food and Drug Administration Staff.

